# Novel Mutations in the *GTPBP3* Gene for Mitochondrial Disease and Characteristics of Related Phenotypic Spectrum: The First Three Cases From China

**DOI:** 10.3389/fgene.2021.611226

**Published:** 2021-07-01

**Authors:** Hui-ming Yan, Zhi-mei Liu, Bei Cao, Victor Wei Zhang, Yi-duo He, Zheng-jun Jia, Hui Xi, Jing Liu, Fang Fang, Hua Wang

**Affiliations:** ^1^Department of Genetic Medicine, Hunan Provincial Maternal and Child Health Care Hospital, Changsha, China; ^2^National Health Commission Key Laboratory of Birth Defect, Research and Prevention, Changsha, China; ^3^Newborn Screening Center of Hunan Province, Changsha, China; ^4^Department of Neurology, National Center for Children’s Health, Beijing Children’s Hospital, Capital Medical University, Beijing, China; ^5^Department of Neonatology, Hunan Provincial Maternal and Child Health Care Hospital, Changsha, China; ^6^Department of Human and Molecular Genetics, Baylor College of Medicine, Houston, TX, United States; ^7^AmCare Genomics Lab, Guangzhou, China

**Keywords:** mitochondrial disease, Combined Oxidative Phosphorylation Deficiency 23, *GTPBP3* gene, hyperlactacidemia, hyperalaninemia

## Abstract

Combined Oxidative Phosphorylation Deficiency 23 (COXPD23) caused by mutations in *GTPBP3* gene is a rare mitochondrial disease, and this disorder identified from the Chinese population has not been described thus far. Here, we report a case series of three patients with COXPD23 caused by *GTPBP3* mutations, from a severe to a mild phenotype. The main clinical features of these patients include lactic acidosis, myocardial damage, and neurologic symptoms. Whole genome sequencing and targeted panels of candidate human mitochondrial genome revealed that patient 1 was a compound heterozygote with novel mutations c.413C > T (p. A138V) and c.509_510del (p. E170Gfs^∗^42) in *GTPBP3*. Patient 2 was a compound heterozygote with novel mutations c.544G > T (p. G182X) and c.785A > C (p.Q262P), while patient 3 was a compound heterozygote with a previously reported mutation c.424G > A (p.E142K) and novel mutation c.785A > C (p.Q262P). In conclusion, we first describe three Chinese individuals with COXPD23, and discuss the genotype-phenotype correlations of *GTPBP3* mutations. Our findings provide novel information in the diagnosis and genetic counseling of patients with mitochondrial disease.

## Introduction

Mitochondrial disease is a clinically and genetically heterogeneous group of disorders that result from dysfunction of the mitochondrial respiratory chain and oxidative phosphorylation (OXPHOS) (Craven et al., 2017). It is an unusual clinical entity with an estimated incidence of 5/100,000 in children and most often affects organs with the highest energy demands such as the brain, heart, liver, and skeletal muscle, etc. (Skladal et al., 2003; Craven et al., 2017; Rahman and Wolf, 2017). Mitochondrial function is under the dual genetic control of both the mitochondrial and nuclear genomes. The human *GTPBP3* gene (MIM #608536) is a nuclear encoded gene and is mapped on chromosome 19p13.11. It encodes the Gtpbp3 protein, a mitochondrial GTP-binding protein involving mitochondrial tRNA (mt-tRNA) post-transcription modification. Defects of *GTPBP3* inhibit taurine modification of mt-tRNA, causing mitochondrial translation defects, leading to Combined Oxidative Phosphorylation Deficiency 23 (OMIM #608536, COXPD23). It is autosomal recessive disease characterized by lactic acidosis, hypertrophic cardiomyopathy and encephalopathy with onset in early childhood (Kopajtich et al., 2014; Murayama et al., 2019). To date, 12 cases of COXPD23 and 15 different mutations in *GTPBP3* have been described (Kopajtich et al., 2014; Murayama et al., 2019), but COXPD23 caused by mutations in GTPBP3 identified from the Chinese population has not been reported. In this report, we describe a case series of COXPD23 cases caused by *GTPBP3* mutations. The mechanism, genotype-phenotype correlations, and diagnosis of this clinical entity are discussed, and the literature is reviewed.

## Materials and Methods

### Next-Generation Sequencing and Bioinformatics Analysis

Blood samples were collected from the probands and family members. According to the manufacturer’s instructions, genomic DNA was extracted using the SolPure Blood DNA kit (Magen) followed by DNA fragmentation using Q800R Sonicator (Qsonica). Based on the paired-end libraries, custom designed NimbleGen SeqCap solution-based exome capture reagent (Roche NimbleGen, Madison, WI) was used for fragments enrichment prior to sequencing on a NextSeq500 sequencer (Illumina, San Diego, CA). Multiple computational algorithms were applied to achieve pathogenicity and evolutionary conservative analysis as previously described (Jiang et al., 2017). Sanger sequencing was used to confirm the variants and determine the family co-segregation state. The interpretation of variants was manipulated according to the American College of Medical Genetics (ACMG) guidelines (Richards et al., 2015). Finally, the interpretation of variants was classified according to the ACMG guidelines, combined with the clinical manifestations and pedigree analysis of the proband. This study was performed in accordance with the Declaration of Helsinki and approved by the Ethical Committees of Beijing Children’s Hospital and Hunan Provincial Maternal and Child Health Care Hospital. Written informed consent was obtained from the children’s parents/guardians.

## Results

### Patient Series Presentation

**Patient 1**, a 40-week gestation male baby, with a birth weight of 3,350 g, was delivered by cesarean section to a 32-year-old mother. His mother had a healthy boy aged 4 years old, and she suffered from Thrombocytopenia during this pregnancy. The infant’s Apgar scores were 10 at 1 min and 9 at 10 min after delivery. Breastfeeding was initiated within 30 min after birth, but the infant demonstrated poor feeding. At the 17th hour after delivery, the infant experienced a sudden onset of dyspnea, cyanosis, mild stridor, and difficulty in suction. Physical examination revealed a temperature of 36°C, respiratory rate of 25/min, pulse rate of 118/min, BP of 66/29 mmHg, and SpO_2_ of 76% (on mechanical ventilation), poor response, severe cyanosis, weak pulse, cool extremities, and low muscle tone. Routine laboratory investigations revealed hyperglycemia (7.3 mmol/L, normal: 3.9–6.1 mmol/L), pronounced hyperlactatemia (26 mmol/L, normal: <12 mmol/L), severe metabolic acidosis (PH 7.098, BE -29 mmol/L). The cerebrospinal fluid (CSF) lactate concentration increased to 20 mmol/L (normal: 0.9–2.7 mmol/L), and blood ketone concentration was 0.6 mmol/L (normal: 0–0.3 mmol/l). Moreover, serum lactate dehydrogenase (LDH) (721.7 U/L, normal: 120–250 U/L), creatine kinase (CK) (3,395.6 U/L, normal: 26–308 U/L), CK-MB (99.7 U/L, normal: 0–24 U/L), and myoglobin (656.2 U/L, normal: 0–90 U/L) levels were markedly elevated, consistent with skeletal muscle damage. The blood count, serum electrolytes, and ammonia level were normal. A chest x-ray showed significant cardiomegaly with a cardiothoracic ratio of 0.7. Urinary organic acid analysis by gas chromatography-mass spectrometry (GC-MS) revealed that urinary lactate concentration was significantly increased. Tandem-mass-spectrometry (MS-MS) showed a grossly increased level of alanine (2,384.51 μmol/L, normal: 100–510 μmol/L). The infant was promptly treated with mechanical ventilation, fluid resuscitation, correct metabolic acidosis, and intravenous supplementation of L-carnitine Coenzyme Q10, vitamin B1, and C. Unfortunately, the infant’s condition deteriorated progressively, and he died of congestive heart failure (CHF) on the 4th day of life.

**Patient 2**, a girl was born to non-consanguineous healthy parents after an uneventful pregnancy. Her mother had one miscarriage at 7 weeks. She had normal developmental milestones before she was 6 months old. At the age of 1 year, she was first noticed to have remarkable developmental delay, with inflexibility of the lower extremities, and inability to stand walk alone, or speak. When she was first admitted to our hospital at the age of 2.5 years, she could not walk for a few steps steadily with wide legs stance and still couldn’t speak, run, or jump. Physical examination revealed slight hypotonia of lower extremities. The routine laboratory test showed plasma lactate was markedly elevated (7.74–14 mmol/L, normal: 0–2.2 mmol/L), and CK-MB was slightly elevated (32 U/L, normal: 0–25 U/L). The plasma ammonia, glucose, LDH, aminotransferases, and CK were all within normal limits. GC-MS showed a grossly increased level of urine lactate. MS-MS revealed hyperalanine of 256.75 μmol/L (normal: 51–180 μmol/L) with normal plasma acylcarnitine profiles. Brain magnetic resonance imaging (MRI) demonstrated bilateral lesions in the midbrain, thalamus and dentate body of the cerebellum. The patient was treated with thiamine, riboflavin, Vitamin C, CoQ10, and carnitine. However, there was no significant improvement in her condition at the 6-month follow-up.

**FIGURE 1:**
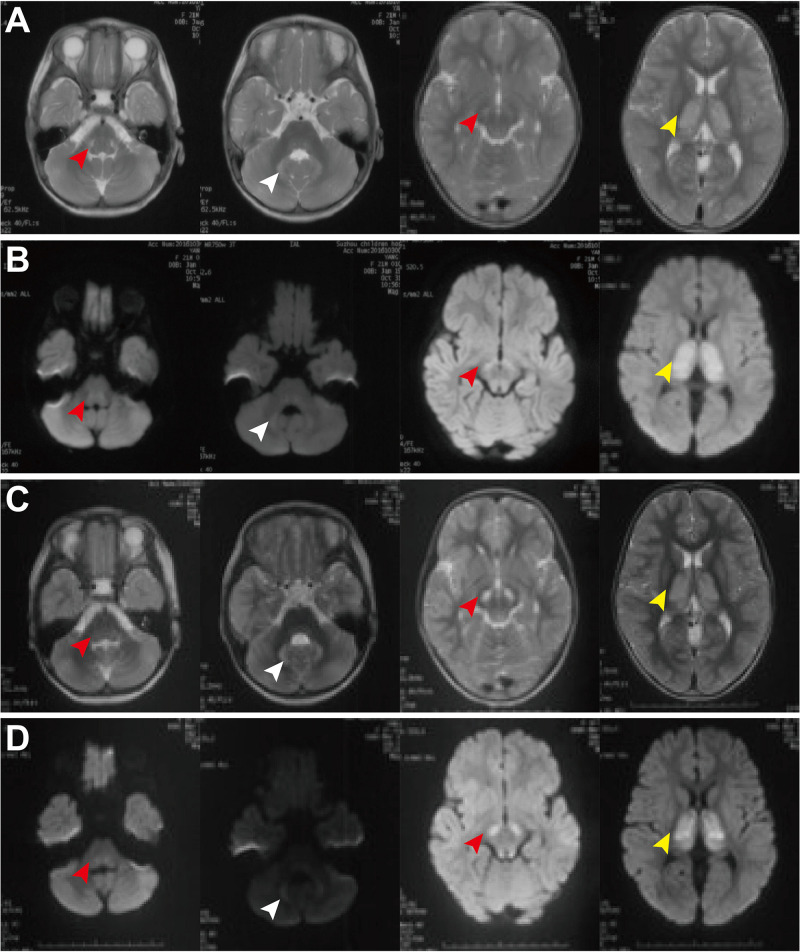
Brain MRI of patient 3 at the ages of 1 year and 9 months old **(A,B)** and 3 years and 8 month old **(C,D)**. MRI demonstrated bilateral lesions in the brain stem (red arrowheads), thalamus (white arrowheads), and dentate body of cerebellum (yellow arrowheads), with diffusion limitation in diffusion weighted image. There is no significant improvement during the follow-up period.

**Patient 3,** was the first child of non-consanguineous healthy parents. She was born at 41 weeks of gestational age after an uneventful pregnancy. At the age of 1 year and 9 months, she was admitted with three episodes of sudden convulsions. She was also found to have mild developmental delay and intellectual disability, fatigability, and hypertrophic cardiomyopathy. At the age of 3 years and 9 months, she could walk and run but easily fell down. She could understand most of the others’ expressions and speak a few words but was unable to construct a complete sentence. Plasma lactate was consistently elevated (4.26–16 mmol/L, normal: <2.2 mmol/L). There were no significant abnormalities in other routine laboratory tests, urine organic acid profile, plasma amino acid, and acylcarnitine profiles. The electroencephalogram (EEG) and electrocardiography (ECG) were normal, but echocardiography revealed left ventricular hypertrophy (no data or images were found regarding this description). Brain MRI demonstrated bilateral lesions in the brain stem, thalamus and dentate body of the cerebellum, with diffusion limitation in diffusion-weighted images (Figure 1). She was treated with thiamine, CoQ10, and carnitine. There was no significant improvement in her condition at the 8-month follow-up.

### Molecular Genetics Analysis

**FIGURE 2:**
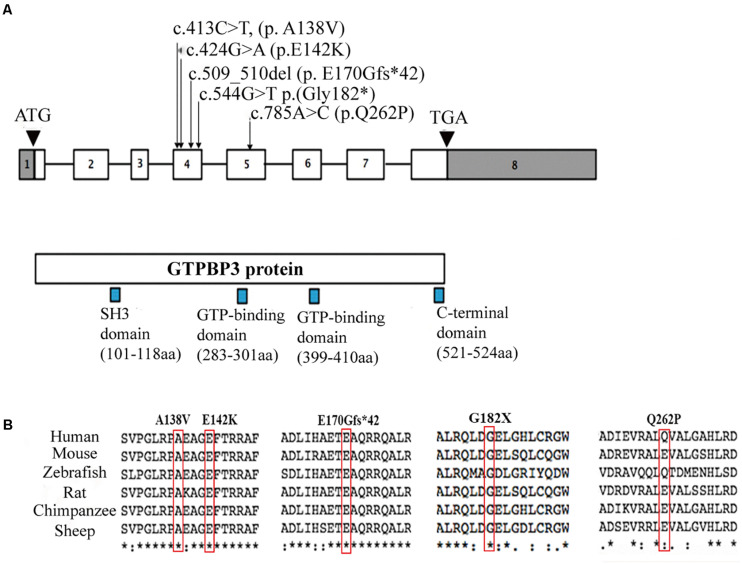
Localizations and amino acids conservative analysis of *GTPBP3* mutations. **(A)** Schematic presentation of *GTPBP3* gene structure and the locus of identified mutations. Square indicates exons and line indicates introns. **(B)** Amino acids conservative analysis of the mutations among species.

Whole genome sequencing (WES) and targeted panels of candidate human mitochondrial genome were provided and revealed five variants in the *GTPBP3* gene in these three Chinese families. The mutations c.413C > T (p. A138V), c.509_510del (p. E170Gfs^∗^42), c.544G > T (p. G182X), and c.424G > A (p.E142K) were localized in exon 4 while the mutation c.785A > C (p.Q262P) was localized in exon 5. The encoded amino acid residues of these mutations are all localized between the SH3 domain (101–118) and GTP-binding domain (283–301) of Gtpbp3 protein (Figure 2A). Further cross-species conservative analysis indicated that Ala138, Glu142, Glu170, and Gly182 residues are all highly conserved during evolution. Despite the amino acid variation at position 262 (Glu or Gln) among the six investigated species, both Glu and Gln are hydrophilic residues, which present high structure similarities (Figure 2B). These mutations cause amino acid alteration at highly conserved residues, which is physiochemically different from how the wild-type residue would impact the spiral structure and the function of the protein.

**FIGURE 3:**
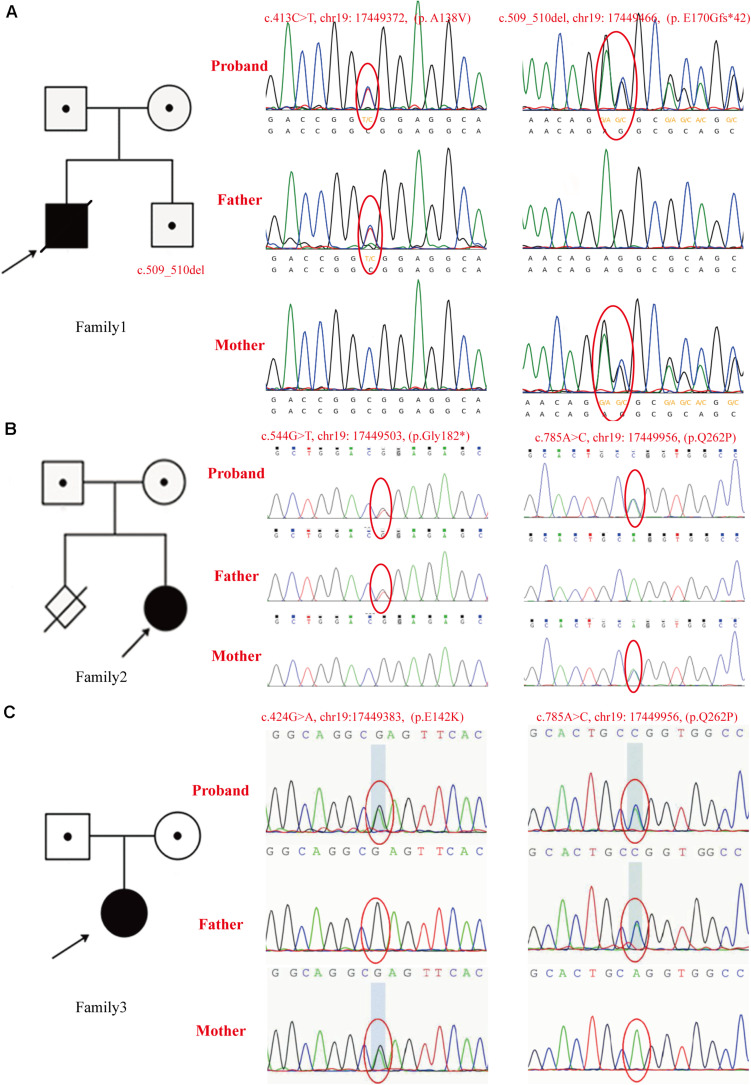
The pedigrees and identified *GTPBP3* mutations. **(A)** In family 1, the c.413C > T (p. A138V) mutation was detected in the proband and his father, c.509_510del (p. E170Gfs*42) was detected in the proband and his mother. As shown in the pedigree of the nuclear family, c.509_510del (p. E170Gfs*42) was found in his brother as validated by Sanger sequencing. **(B)** Mutations identified in family 2 as confirmed by Sanger sequencing. The c.544G > T (p. G182X) mutation was found in the proband and her father, and the c.785A > C (p.Q262P) mutation was found in the proband and her mother. As shown in the pedigree of the nuclear family, there is a history of firstborn spontaneous abortion of the mother at the 7th week of gestation. **(C)** Mutations found in family 3 as confirmed by Sanger sequencing. The c.785A > C (p.Q262P) was found in the proband and her father, mutation c.424G > A (p.E142K) was found in the proband and her mother. The arrow indicates the proband.

Patient 1 was a compound heterozygote with novel mutations c.413C > T (p. A138V) and c.509_510del (p. E170Gfs^∗^42) in *GTPBP3*. Sanger sequencing revealed that the c.413C > T (p. A138V) was inherited from the paternal allele while the c.509_510del (p. E170Gfs^∗^42) came from the maternal allele. Additionally, the infant’s brother was a carrier of the mutation c.509_510del (p. E170Gfs^∗^42), which was inherited from his mother (Figure 3A). The mutation c.413C > T (p. A138V) was not found in ExAc, dbSNP, 1,000 G, or ESP, and mutation c.509_510del (p. E170Gfs^∗^42) is less frequent in our reference population genetic database (Table 1, PM2). Furthermore, the mutation c.413C > T (p. A138V) was predicted to be pathogenic by multiple computational analyses (Table 1, PP3). For recessive disorders, the frameshift mutation c.509_510del (p. E170Gfs^∗^42) in *GTPBP3* (Table 1, PVS1) was detected in trans with a pathogenic variant (Table 1, PM4). And the newborn’s phenotype (hyperlactacidemia and metabolic acidosis complicated with respiratory acidosis) is highly specific for a disease with a single genetic etiology (Table 1, PP4). Given the above analysis, we speculate that compound heterozygote mutations c.413C > T (p. A138V) and c.509_510del (p. E170Gfs^∗^42) mutations in *GTPBP3* are the cause for COXPD23 with recessive inheritance (Table 1).

**TABLE 1:** The molecular genetics analysis of three patients.

Patient ID	Variant (paternal)	Variant (maternal)	Evidence of pathogenicity	ACMG classification
#1	c.413C > T, chr19: 17449372, (p. A138V)		PM2 + PM3 + PP3 + PP4	Likely pathogenic
		c.509_510del, chr19: 17449466, (p. E170Gfs*42)	PVS1 + PM2	Likely pathogenic
#2	c.544G > T, chr19: 17449503, (p.Gly182*)		PVS1 + PM2	Likely pathogenic
		c.785A > C, chr19: 17449956, (p.Q262P)	PM2 + BP4	Uncertain significance
#3	c.785A > C, chr19: 17449956, (p.Q262P)		PM2 + BP4	Uncertain significance
		c.424G > A, chr19:17449383, (p.E142K)	PM2 + PP3	Uncertain significance

**Trio whole exome sequencing (WES) successfully defined GTPBP3 gene variations in chromosome 19 (GRCh37/hg19, NM_133644). Evidence of Pathogenicity were according to ACMG standards and guidelines 10.1038/gim.2015.30. And the missense prediction is analyzed on the website http://varcards.biols.ac.cn/.**

Patient 2 was a compound heterozygote with novel mutations c.544G > T (p. G182X) and c.785A > C (p. Q262P) in *GTPBP3*, and the c.544G > T (p. G182X) was inherited from the paternal allele, while the c.785A > C (p. Q262P) came from the maternal allele (Figure 3B). The frameshift mutation [c.544G > T (p. G182X)] (Table 1, PVS1) was not found in ExAc, dbSNP, 1,000 G, or ESP, and missense mutation [c.785A > C (p. Q262P)] is less frequent in our reference population genetic database (Table 1, PM2). In addition, the results of bioinformatics analysis of missense mutation [c.785A > C (p. Q262P)] were inconsistent. PolyPhen-2 and SIFT predicted that it was benign, but Mutation-Taster predicted that it was harmful (Table 1, BP4). However, the patient’s clinical manifestations (developmental delay, fatigability, and hyperlactacidemia) are highly consistent with COXPD23 caused by *GTPBP3* defects. Therefore, we speculate that compound heterozygote mutations c.544G > T (p. G182X) and c.785A > C (p. Q262P) mutations in *GTPBP3* are the cause for COXPD23 with recessive inheritance (Table 1).

Patient 3 was a compound heterozygote with c.424G > A (p. E142K) and novel mutation c.785A > C (p.Q262P) in *GTPBP3*, and the c.785A > C (p.Q262P) was inherited from the paternal allele while the c.424G > A (p.E142K) from the maternal allele (Figure 3C). The mutation c.424G > A (p. E142K) was not found in ExAc, dbSNP, 1,000 G, or ESP, and missense mutation [c.785A > C (p. Q262P)] is less frequent in our reference population genetic database (Table 1, PM2). The region of variation [c.424G > A (p. E142K)] is an important part of the Gtpbp3 protein, and the amino acid sequences of different species are highly conserved. Computer-aided analysis predicts that this variation is more likely to affect the structure and function of proteins (Table 1, PP3). Considering the continuous elevation of plasma lactic acid and the involvement of multiple systems, we speculate that compound heterozygote with mutation c.424G > A (p. E142K) and the novel mutation c.785A > C (p.Q262P) in *GTPBP3* are the cause for COXPD23 with recessive inheritance (Table 1).

## Discussion

In this report, we described three cases of COXPD23 caused by mutations in *GTPBP3* from Chinese population, one of them (patient 1) with severe neonatal presentation and a fatal outcome, carrying the novel mutations c.413C > T (p. A138V) and c.509_510del (p. E170Gfs^∗^42) in compound heterozygotes, and the remaining two (patients 2 and 3) with a mild presentation characterized by developmental delay with no significant improvement of the clinical condition during follow-up. Patient 2 was a compound heterozygote with novel mutations c.544G > T (p. G182X) and c.785A > C (p.Q262P), while patient 3 was a compound heterozygote with c.424G > A (p.E142K) and novel mutation c.785A > C (p.Q262P).

The post-transcriptional modification is essential to the maturation processes of tRNAs to form the cloverleaf structure resulting in a stable and correctly functioning tRNAs, and a variety of modified nucleosides are required in these processes (Van Haute et al., 2015). The 5-taurinomethyluridine (τm^5^U) modification, identified in mt-tRNALeu^(UUR)^ and mt-tRNA^*Trp*^, is required for accurate decoding of purine-ending codons and prevent misreading of pyrimidine-ending codons, thereby promoting efficient decoding of cognate codon (Suzuki and Suzuki, 2014). Human *GTPBP3* gene encoding the mitochondrial GTP-binding protein Gtpbp3, has been documented to be responsible for the τm^5^U synthesis, which involved in the post-transcriptional modification of mt-tRNALeu^(UUR)^ and mt-tRNA^*Trp*^ (Li and Guan, 2002). A study by Asano et al. (2018) demonstrated that τm^5^U was absent from mt-tRNALeu^(UUR)^ and mt-tRNA^*Trp*^ in *GTPBP3* KO cells. *GTPBP3* KO cells exhibited marked reductions in oxygen consumption rate (OCR) and mitochondrial translation, reduced levels of complex I subunit proteins and deficient complex I activity. Their findings demonstrated that repression of mitochondrial translation due to lack of m^5^U34 in *GTPBP3* KO might disrupt maturation and stability of respiratory chain complexes (Asano et al., 2018). Villarroya et al. (2008) found that in comparison with the negative controls, the human *GTPBP3*-silenced cells exhibited significant mitochondrial dysfunction, manifested by a significant decrease of OCR and mitochondrial membrane potential, a 40% (±9) decrease in ATP levels, as well as a 28% (±3) increase in mitochondrial superoxide generation. Taken together, these studies documented that the *GTPBP3* gene is associated with mt-tRNAs post-modification and defects of *GTPBP3* will lead to mitochondrial dysfunction. In addition, Boutoual et al. (2018) reported that stable silencing of *GTPBP3* triggers an AMPK-dependent retrograde signaling pathway, which downregulates the mitochondrial pyruvate carrier (MPC), while upregulating the expression of the uncoupling protein 2 (UCP2) and genes involved in glycolysis and fatty acid oxidation, leading to an uncoupling of glycolysis and oxidative phosphorylation. The uncoupling is expected to result in lactic acidosis, a common clinical trait presents in *GTPBP3* patients.

The association between *GTPBP3* mutations and mitochondrial disease was first described in 2014. Kopajtich et al. (2014) reported 11 individuals from 9 families, carrying homozygous or compound heterozygous mutations in *GTPBP3*. These individuals presented with lactic acidosis, hypertrophic cardiomyopathy, and encephalopathy, consistent with the impaired activity of respiratory complexes (I and IV), and defective translation of mitochondrial proteins, providing strong evidence for the pathological role of mutant *GTPBP3* in mitochondrial disease. Elmas et al. (2019) also described a 10-year- old girl with *GTPBP3* mutations, characterized by early childhood onset of mental motor retardation, seizure, hearing disability, and delayed myelination. These 12 patients came from five ethnic groups, including Romanian, Turkish, Arab, Indian, and Japanese, sharing some similar phenotypes, which might be classified into two forms. The first is the severe form, usually presented within the period of infancy and characterized by acute metabolic decompensation with a rapid deterioration, often presenting with CHF, severe hyperlactacidemia, and consequently early death. Six mutants were identified in this form, including c.484G > C, c.673G > A, c.1009G > C, c.665-2delA, c.424G > A, and c.32_33delinsGTG, causing the loss of TrmE-type G domain, the missense *GTPBP3* variants in TrmE-type G domain, or missense variant in N-terminus of Gtpbp3 protein. The second is the mild form with encephalopathy, cardiomyopathy, and hyperlactacidemia, usually presented in early childhood though it may survive into the second decade. Seven mutants were identified in this form, including c.1291dupC, c.1375G > A, c.476A > T, c.770C > A, c.8G > T, c.934_957del, and c.932C > T, while mutant c.964G > C was observed in both severe and mild forms. These causative mutations mainly concentrate on the C-terminus and N-terminus, and only three carry missense variants in the G domain (Table 2). Our three cases were born to unrelated parents of Chinese origin. Our patient 1 presented with a severe form, with remarkably hyperlactacidemia, metabolic acidosis, and progressive heart damage and died of CHF on the 4th day of life. Patients 2 and 3 presented with a mild form, with developmental delay, fatigability, and hyperlactacidemia. The brain MRI preformed in patients 2 and 3 and showed an involvement of the thalamus, brainstem, and cerebellum, resembling the findings in Leigh syndrome. The clinical status of these two patients is stable and did not show improvement during their follow-up period. Additionally, a grossly increased level of serum alanine was detected in patient 1, along with a mild hyperalaninemia in patient 2. Of note, the hyperalaninemia has not been described in previously reported cases of COXPD23. The elevation in amino acids such as alanine, glycine, proline, and threonine is due to the altered redox state created by respiratory chain dysfunction, and the notable elevation usually occurs during the course of clinical deterioration (Parikh et al., 2015). The hyperalaninemia accompanied by hyperlactacidemia is an important clue for clinical suspicion of mitochondrial disorders, and the marked hyperalaninemia might serve as a red flag for the severe form of mitochondrial diseases.

**TABLE 2:** Clinical and Genetic Findings in Patients with *GTPBP3* Mutations.

Patient ID	Sex	Ethic origin	Onset age	Presenting symptoms	Plasma lactate	TTE	Brain MRI	Outcomes, follow-up time, cause of death	*GTPBP3* mutations	chromosomal position (hg19)	Domain/site
**Severe form**											
#72425 (Kopajtich et al., 2014)	F	No data	3.5 months	Poor feeding, failure to thrive, hypoactivity	23.3 mmol/l	DCM	No data	Died, 8 months, CHF	c.[484G > C; 673G > A; 964G > C], p.[Ala162Pro; Glu225Lys; Ala322Pro]	g.[17449443;1 7449940;1745 0398]	Ala162Pro and Glu225Lys at position between N-terminus and TrmE-type G domain; Ala322Pro at TrmE-type G domain (249–416)
#75191 (Kopajtich et al., 2014)	F	No data	Birth	Poor feeding, hypotonic, respiratory failure	23 mmol/l	HCM	No data	Died, 1 day, asystolia	c.[1009G > C; 1009G > C], p.[Asp337His; Asp337His]	g.[17451887]	Asp337His at TrmE-type G domain (249–416)
#76671 (Kopajtich et al., 2014)	M	No data	Birth	Poor feeding, hypotonic, CHF, metabolic acidosis	5.2 mmol/l	HCM	Bilateral hyperintensities in thalamus	Died, 10 months, CHF	c.[665-2delA; 665-2delA], p.[Ala222Gly; Asp223_Ser270del; Ala222Gly; Asp223_Ser270del]	g.[17449930]	N/A
#81471 (Kopajtich et al., 2014)	M	Romanian	4 weeks	Hypothermia, recurrent apnea metabolic acidosis	11 mmol/l	HCM	Hyperintensities in subthalamic nuclei	Died, 5 weeks, acidosis	c.[424G > A; 424G > A], p.[Glu142Lys; Glu142Lys]	g.[17449383]	Glu142Lys at GTP-binding protein TrmE N-terminus (35–152)
#83904 (Kopajtich et al., 2014)	F	Turkish	1 week	Cardiogenic shock, metabolic acidosis	20 mmol/l	DCM	No data	Died, 9 months, CHF	c.[32_33delinsGTG; 32_33delinsGTG], p.[Gln11Argfs*98; Gln11Argfs*98]	g.[17448452_1 7448453]	Gln11Argfs*98 at transit peptide (1–81)/GTP-binding protein TrmE N-terminus (35–152)
#83905 (Kopajtich et al., 2014)	F	Turkish	Birth	Cardiogenic shock, metabolic acidosis	No data	DCM	No data	Died, 10 days, CHF	c.[32_33delinsGTG; 32_33delinsGTG], p.[Gln11Argfs*98; Gln11Argfs*98]	g.[17448452_1 7448453]	Gln11Argfs*98 at transit peptide (1–81)/GTP-binding protein TrmE N-terminus (35–152)
Our Patient #1	M	Chinese	17 h	Hypothermia, poor response, respiratory failure, cardiogenic shock, metabolic acidosis	26 mmol/l	Normal	No data	Died, 5 days, CHF	c.[413C > T; c.509_510del], p.[Ala138Val; Gln170Glyfs*42]	g.[17449372;17 449468_174494 69delAG]	Ala138Val at GTP-binding protein TrmE N-terminus (35–152); Gln170Glyfs*42 localized between the SH3 domain (101–118) and GTP-binding domain (283–301)
**Mild form**											
#49665 (Kopajtich et al., 2014)	M	Arab	10 years	Intellectual disability, fatigability, visual impairment, slight dyspnea with climbing stairs	3∼7 mmol/l	HCM	Lactate peaks in parietal and precentral cortex	Alive, 14 years	c.[1291dupC; 1375G > A], p.[Pro430Argfs*86; Glu459Lys]	g.[17452324;1 7452408]	C-terminal
#36349 (Kopajtich et al., 2014)	M	Arab	No data	Intellectual disability, fatigability, visual impairment, slight dyspnea with climbing stairs	No data	HCM	Lactate peaks in parietal and precentral cortex	Alive, 17 years	c.[1291dupC; 1375G > A], p.[Pro430Argfs*86; Glu459Lys]	g.[17452324;17 452408]	C-terminal
#66143 (Kopajtich et al., 2014)	M	Arab	2 years	Sudden respiratory failure, CHF	No data	HCM	No data	Alive, 5 years	c.[476A > T; 964G > C], p.[Glu159Val; Ala322Pro]	g.[17449435;17 450398]	Glu159Val at position between N-terminus and TrmE-type G domain; Ala322Pro at TrmE-type G domain (Switch II region (302–312, 314–328)
#75168 (Kopajtich et al., 2014)	F	Indian	2 years	Developmental delay, epileptic seizures	>10 mmol/l	No data	Bilateral hyperintensities in thalamus	Alive, 5 years	c.[770C > A; 770C > A], p.[Pro257His; Pro257His]	g.[17450037]	Pro257His at GTP binding region (256–263); Pro257His at TrmE-type G domain (249–416)
#82790 (Kopajtich et al., 2014)	F	Japanese	1 year	Developmental delay, epileptic seizures, hypotonia	5.7∼6.5 mmol/l	Normal	Bilateral hyperintensities in thalamus	Alive, 2 years	c.[8G > T; 934_957del], p.[Arg3Leu; Gly312_Val319del]	g.[17448428;17 450368_17 450391]	Arg3Leu at transit peptide (1–81); Gly312_Val319del at TrmE-type G domain (249–416); Gly312_Val319del at Switch II region (302–312, 314–328)
Patient No. 24 (Elmas et al., 2019)	F	Turkish	3 weeks	Mental motor retardation, seizure, hearing disability, thrombocytopenia	No data	Normal	Delayed myelination	Alive, 10years	c.[932C > T,932C > T], p.[Pro311Leu, Pro311Leu]	g.[17450270;17 450270]	Pro311Leu at TrmE-type G domain (249–416)
Our Patient #2	F	Chinese	1 year	Developmental delay, hypotonia	7.7∼14 mmol/l	No data	Bilateral lesions in brain stem, thalamus and cerebellum	Alive, 3 years	c.[544G > T; c.785A > C], p.[Gly182X; Gln262Pro]	g.[17449503;17 449956]	Gly182X at position between N-terminus and TrmE-type G domain which cause the loss of TrmE-type G domain and C terminal; Gln262Pro at GTP binding region (256–263)
Our Patient #3	F	Chinese	1 year	Developmental delay, intellectual disability, fatigability,	4.26∼16 mmol/l	HCM	Bilateral lesions in brain stem, thalamus and cerebellum	Alive, 3 years	c.[424G > A; c.785A > C], p.[Glu142Lys; Gln262Pro]	g.[17449383;17 449956]	Glu142Lys at GTP-binding protein TrmE N-terminus (35–152); Gln262Pro at GTP binding region (256–263)

**CHF, congestive heart failure; DCM, dilated cardiomyopathy; HCM, hypertrophic cardiomyopathy; N/A, not applicable; TTE, transthoracic echocardiography.**

Diagnosis of mitochondrial disease is challenging. The next-generation sequence (NGS) is recommended to accurately confirm mitochondrial disease. Two options are currently being implemented—WES and targeted panels of candidate genes including both mt-DNA and known pathogenic nDNA genes (Wong, 2013; Alston et al., 2017). In our patients, we implemented WES and targeted panels of candidate mitochondrial gene and identified five heterozygous rare mutations in *GTPBP3*, among which mutation c.424G > A (p. E142K) was reported previously (Table 2, patient #81471). The other four mutations were novel, including c.413C > T (p. A138V) and c.509_510del (p. E170Gfs^∗^42) in patient 1, c.544G > T (p. G182X), c.785A > C (p.Q262P), and c.785A > C (p.Q262P) in patients 2 and 3. Mutation c.413C > T (p. A138V) causes Ala to Val at GTP-binding protein TrmE N-terminus of Gtpbp3 protein, and c.509_510del (p. E170Gfs^∗^42) causes a frame shift that leads to no translation of GTP domains thereafter. The GTP binding domain participates in binding of guanine nucleotides and Mg^2+^, hydrolysis of GTP or regulates the functional state of GTPases through conformational alteration (Villarroya et al., 2008). Mutations c.544G > T (p. G182X), c.785A > C (p.Q262P) and c.785A > C (p.Q262P) in patients 2 and 3 cause the loss of TrmE-type G domain and C terminal or an impaired GTP domain. Interestingly, the impaired GTP domains were also observed in 9 of 12 previously reported cases with both severe and mild forms (Table 2). Therefore, we speculate that mutations in GTP binding domain may cause different degrees of Gtpbp3 functional impairment.

In conclusion, we first report three Chinese individuals with COXPD23 caused by mutations in *GTPBP3* from severe to mild phenotype. Apart from a previously reported mutation c.424G > A (p. E142K), four novel mutations c.413C > T (p. A138V), c.509_510del (p. E170Gfs^∗^42), c.544G > T (p. G182X) and c.785A > C (p.Q262P) were identified. Our findings provide novel information in the molecular diagnosis and genetic counseling of patients with COXPD23. Admittedly, the sample size is small, and more in-depth investigations are needed to further elucidate the genotype-phenotype correlations in COXPD23.

## Data Availability Statement

The data presented in the study are deposited in the (GSA-Human) repository (https://bigd.big.ac.cn/gsa-human/browse/HRA000939), accession number (HRA000939).

## Ethics Statement

The studies involving human participants were reviewed and approved by the Ethical Committees of Beijing Children’s Hospital and Hunan Provincial Maternal and Child Health Care Hospital. Written informed consent to participate in this study was provided by the participants’ legal guardian/next of kin.

## Author Contributions

H-MY and Z-ML participated in the treatment for the patients, analyzed, and interpreted the data, acquired the literature data, wrote the manuscript, and created Table 2. BC cared for the patients, provided clinical data, and performed clinical assessments. VZ and Y-DH performed genetic analysis, reviewed the data, and helped to revise the manuscript for molecular genetics analysis content. Z-JJ, HX, and JL reviewed the data and helped to draft the manuscript. FF and HW cared for the patients, performed clinical assessments, revised the final version of the manuscript, and supervised the study. All the authors reviewed the final version of the manuscript.

## Conflict of Interest

The authors declare that the research was conducted in the absence of any commercial or financial relationships that could be construed as a potential conflict of interest.
